# Implementation of community case management of malaria in malaria endemic counties of western Kenya: are community health volunteers up to the task in diagnosing malaria?

**DOI:** 10.1186/s12936-022-04094-w

**Published:** 2022-03-05

**Authors:** Enock Marita, Bernard Langat, Teresa Kinyari, Patrick Igunza, Donald Apat, Josephat Kimori, Jane Carter, Richard Kiplimo, Samuel Muhula

**Affiliations:** 1 Amref Health Africa, P. O. Box 30125, Nairobi, 00100 Kenya; 2 University of Nairobi, P.O Box 30197, Nairobi, 00100 Kenya; 3 National Tuberculosis, Leprosy and Lung Disease Programme, P.O. Box 20781, Nairobi, Kenya

**Keywords:** Community case management of malaria, Community health volunteers, Test positivity rate, Rapid diagnostic test

## Abstract

**Background:**

Community case management of malaria (CCMm) is an equity-focused strategy that complements and extends the reach of health services by providing timely and effective management of malaria to populations with limited access to facility-based healthcare. In Kenya, CCMm involves the use of malaria rapid diagnostic tests (RDT) and treatment of confirmed uncomplicated malaria cases with artemether lumefantrine (AL) by community health volunteers (CHVs). The test positivity rate (TPR) from CCMm reports collected by the Ministry of Health in 2018 was two-fold compared to facility-based reports for the same period. This necessitated the need to evaluate the performance of CHVs in conducting malaria RDTs.

**Methods:**

The study was conducted in four counties within the malaria-endemic lake zone in Kenya with a malaria prevalence in 2018 of 27%; the national prevalence of malaria was 8%. Multi-stage cluster sampling and random selection were used. Results from 200 malaria RDTs performed by CHVs were compared with test results obtained by experienced medical laboratory technicians (MLT) performing the same test under the same conditions. Blood slides prepared by the MLTs were examined microscopically as a back-up check of the results. A Kappa score was calculated to assess level of agreement. Sensitivity, specificity, and positive and negative predictive values were calculated to determine diagnostic accuracy.

**Results:**

The median age of CHVs was 46 (IQR: 38, 52) with a range (26–73) years. Females were 72% of the CHVs. Test positivity rates were 42% and 41% for MLTs and CHVs respectively. The kappa score was 0.89, indicating an almost perfect agreement in RDT results between CHVs and MLTs. The overall sensitivity and specificity between the CHVs and MLTs were 95.0% (95% CI 87.7, 98.6) and 94.0% (95% CI 88.0, 97.5), respectively.

**Conclusion:**

Engaging CHVs to diagnose malaria cases under the CCMm strategy yielded results which compared well with the results of qualified experienced laboratory personnel. CHVs can reliably continue to offer malaria diagnosis using RDTs in the community setting.

## Background

Early detection and treatment of malaria contributes to reduced complications and deaths [1]. A mixed approach to providing health services at both health facility and community levels is appropriate where only about 70% of people in sub-Saharan Africa use public facilities as the first point of care when a family member has fever [1].

Globally, given the limited human resources in the health sector, the community-based approach has been promoted as a cost-effective and pro-poor intervention to improve the accessibility of healthcare [1–3]. This underscores the importance of community health volunteers (CHVs) as a key element in the community-based approach to most populations in low- and middle-income countries [4]. CHVs are generally defined as non-professional, lay health workers who work in the communities where they reside, and who are equipped with training and incentives to provide promotional, preventive and basic curative healthcare services to community members [5, 6].

In Kenya, CHVs are recruited at community meetings (*barazas*) called by area leaders or community health committees (CHC), using set criteria [7]. CHVs are organized into community units (CUs) supervised by community health extension workers (CHEWs). Each CHV provides services to an average of 100 households, linking them to the formal health sector; about 5,000 community members are served by CHVs within each community unit [8].

Community case management of malaria (CCMm) is an equity-focused strategy that complements and extends the reach of health services by providing timely and effective diagnosis and treatment to populations with limited access to facility-based healthcare [9]. In Kenya, the CCMm strategy utilizes CHVs who have received training on performing and interpreting malaria rapid diagnostic tests (RDT), and prescribe artemether lumefantrine (AL) to confirmed, uncomplicated malaria cases. CHVs refer pregnant women with suspected malaria, suspected severe malaria cases, patients with negative malaria test results, and patients with persistent symptoms to health facilities for further management.

In western Kenya, a malaria-endemic zone, CCMm has been adapted increasingly since 2012 as an approach to increase timely access to malaria care and treatment. About 7,420 CHVs have been trained and equipped with health commodities and tools to promptly diagnose and treat uncomplicated malaria cases at community level and help prevent progression to severe life-threatening disease. CHVs receive 3 days’ training with theory and practical sessions, and thereafter receive on-the-job training during supervision and monthly meetings by CHEWs.

CHVs are also trained to identify severe malaria cases for early referral and thus help to reduce malaria deaths. They hold monthly meetings to discuss their reports and progress, and learn from each other under the leadership of the CHEW. The CHEW also performs on-site support supervision to ensure CHVs are providing quality servicse to the community, including the performance of RDTs. While on site, CHEWs observe how CHVs perform and intrepret RDTs and take corrective action as required. Any of these corrective actions and good performances are discussed during monthly meetings to ensure the CHVs are informed to promote improvemed performance.

CHVs are part of the first level of national malaria monitoring, and conduct epidemiological surveillance of malaria cases at community level. CHVs submit monthly reports to the Kenya Health Information System (KHIS) and thus contribute to the national malaria control strategy with up-to-date information [10].

Symptom-based malaria diagnosis is inaccurate and contributes to poor management of febrile illness, over-treatment of malaria, and may promote drug resistance to current anti-malarial drugs [11]. The World Health Organization (WHO) recommends testing of all suspected malaria cases before treatment as best practice in malaria case management [12]. The 2019–2023 Kenya Malaria Strategy emphasizes this recommendation with testing in healthcare facilities using microscopy and malaria RDT [13]. While microscopy is the diagnostic test of choice in health facilities with laboratories, RDTs are used in facilities where microscopy is unavailable due to several factors, such as lack of microscopes, trained laboratory personnel or electricity. Testing of malaria in a community setting is entirely by RDT with the intention of reducing the practice of presumptive malaria treatment and irrational use of anti-malarial treatment drugs.

While microscopy detects the presence of malaria parasites in blood by direct observation, RDT detects the presence of circulating malaria parasite antigens. The most commonly used RDT detects *Plasmodium falciparum*-specific histidine-rich protein 2 (PfHRP2) while others detect lactate dehydrogenase (LDH) and aldolase. RDT results may remain positive for a variable amount of time (5–61 days) following effective treatment with anti-malarial drugs, depending on the type of RDT used, age and treatment, thereby affecting their specificity [14]. Sensitivity is associated with the inherent performance of the test, as well as quality issues related to handling of test kits and performance of the testing procedure. Although CHVs undergo training on the use of RDT, storage and transport conditions and human error may affect the validity of the test results. Procedural factors include the quality of the blood drop as well as the time taken by the operator to read the test results [15].

The National Malaria Control Programme uses routine surveillance data reported in the KHIS to produce a quarterly Malaria Surveillance Bulletin. In the July–September 2018 issue, the all-age malaria test positivity rate (TPR) was 24% with the TPR in the malaria-endemic lake zone being comparatively high at 35% [16]. However, these data are not disaggregated to facilities or community level. From the CCMm routine data reported for the same period, the average TPR for malaria RDTs performed by CHVs in the malaria-endemic lake zone was almost two-fold at 67%. There was therefore a need to evaluate the performance of CHVs in conducting RDTs and determine the accuracy of their reports in comparison with tests performed by qualified laboratory personnel.

## Methods

The study was conducted in Kakamega, Vihiga, Siaya and Migori Counties, in the malaria-endemic lake zone in Kenya, between September and October 2020. The study population was selected from CHVs conducting CCMm in these counties. The climate in this area is mainly tropical, with variations due to altitude, and rainfall all year round with warm temperatures that influence mosquito populations and malaria transmission. The main sources of livelihood are agriculture, small-scale businesses and fishing. There are about 9,000 CHVs covering about 30% of the population and 385 public health facilities in the four counties [17].

This was a cross-sectional survey to evaluate the performance of CHVs in testing for malaria using RDTs. A quantitative sub-set of the study data was used where 200 CHVs were observed conducting RDT on 200 patients. These results were then compared with results from RDTs performed by experienced medical laboratory technicians (MLTs) using a second sample of capillary blood from the same patient. Blood films were also prepared and examined in the laboratory setting by Level 1 microscopists (as defined by WHO competency levels) [18] as back-up verification as required. In cases with discordant results, the RDT results of the MLTs were used to manage patients.

Multi-stage cluster sampling was used to select the study sample, with the sampling frame as the eight malaria-endemic counties, sub-counties and CUs where CCMm is practised. The first stage involved random sampling of four counties based on their predominantly cultural backgrounds. The sample was then proportionately apportioned to the four counties based on the number of CUs implementing CCMm. The second stage was a random selection of sub-counties from each randomly selected county, followed by a random selection of CUs from the selected sub-counties. Consecutive sampling was then used to identify five CHVs who had encountered a suspected malaria case from a sampled CU, that is, from a sampled CU, only five CHVs were observed.

Research assistants (RAs) were trained on study procedures before starting data collection. CHVs tested patients presenting with symptoms and signs of malaria; consenting patients with suspected uncomplicated malaria were included in the study. Patients with suspected severe or complicated malaria, pregnant women and children under one year old were excluded. The RDT brand used was CareStart Malaria™ (AccessBio, USA), obtained from government central stores using the usual procedures; storage and handling strictly met the manufacturer’s guidelines.

MLTs independently performed an RDT (same brand and batch number as used by the CHV) on the same participant by performing a second prick to collect capillary blood. MLTs also prepared thick and thin blood films (on the same slide) for back-up microscopy. The CHVs and MLTs were blinded to the results of each other. Thin films were fixed in methanol and air-dried, then both thick and thin films were stained with 10% Giemsa solution for 15 min. Staining was done within 12 h to avoid auto-fixation of films. Malaria parasites were recorded as the number of asexual parasites counted per 500 white blood cells in the thick film. If no parasite was found in at least 100 fields at 1000 magnification in the thick film, the result was recorded as no malaria parasites seen. All thick and thin blood films were read by two WHO-certified, Level 1 microscopists and any discrepancy resolved by a third Level 1 microscopist. All microscopists were blinded to the results of the malaria RDTs.

For ease of tracking and analysis, the study participants were given unique identifier numbers containing the CU code, CHV code and study subject number. The RDT strip and blood slides were labelled with the same unique identifier numbers. A log with a record of the RDT results (from the CHV and MLTs) and blood slides were maintained using the identifier number; only the study coordinator had access to this log to ensure the blinding of test results.

Data were captured using electronic Open Data Kit (ODK). The log data kept by the RAs were entered at the end of each day into ODK using a tablet. The data were transmitted to a server hosted by Amref Health Africa in real-time. At the end of each study day, data transmitted to the server were reviewed by the study coordinator and any quality issues flagged for immediate correction. The data were stored on a password-protected computer with back-up on a password-protected external hard-drive only accessible to authorized study staff. Quantitative data were downloaded from the server into Excel (Microsoft, USA) and transferred to STATA version 15 (Stata-Corp, College Station, TX, USA) for statistical analysis.

Cohen’s kappa statistic for inter-rater reliability testing was calculated to establish the level of agreement between the CHVs and qualified laboratory staff. The kappa statistic (or kappa coefficient) was used to assess the strength of the agreement. Interpretation of kappa was as follows:  < 0.20 slight agreement, 0.21–0.40 fair agreement, 0.41–0.60 moderate agreement, 0.61–0.80 substantial agreement, and 0.81–0.99 almost perfect agreement. Inter-reader agreement for facilities versus reference values was expressed as kappa (κ) with a 95% confidence interval (CI) using the ‘kapci’ function in Stata [19].

Sensitivity and specificity were calculated as the proportion of RDT-positive and -negative test results obtained by the CHVs against the results of the MLTs. Positive and negative predictive values were calculated as the proportion of true positive results among all positive samples and the proportion of true negative results among all negative samples, respectively.

## Results

A total of 200 CHV participants were enrolled into the study, distributed proportionate to the size of each county, with 42% from Kakamega, 27% from Migori, 18.5% from Siaya, and 12.5% from Vihiga Counties. The socio-demographic characteristics of the study participants are shown in Table 1. The CHVs’ median age was 45 (IQR: 43–47) years, age range 26–75 years.

**Table 1:** Socio-demographic characteristics of the study participants

Characteristic	Category	Overall
Gender	Male	57 (28.5%)
	Female	143 (71.5%)
Age group (years)	26–35	36 (18%)
	36–45	64 (32%)
	46–55	67 (33%)
	56–65	29 (14%)
	66 +	4 (2%)
Years of experience	< 1	14 (7%)
	1–2	76 (38%)
	3–5	43 (21%)
	> 5	49 (24%)
	Missing	18 (9%)
Marital status	Single	3 (1.5%)
	Married	174 (87%)
	Widowed	23 (11.5%)
Education level	Completed primary education	47 (23.5%)
	Some primary education	18 (9%)
	Secondary education	62 (31%)
	Some secondary education	63 (31.5%)
	College	10 (5%)
Religion	Christian	194 (97%)
	Muslim	6 (3%)

At 95% CI (0.82, 0.95), the kappa score was 0.89 indicating almost perfect agreement (92.5%) in RDT results between CHVs and MLTs. The standard error was 0.07 and Prob > Z was 0.000.

The overall sensitivity and specificity between CHVs and MLTs were 95.0% (95% CI 87.7, 98.6) and 94.0% (95% CI 88.0, 97.5), respectively (Table 2). The sensitivity for male and female CHVs was 100% (95% CI 83.9, 100) and 93.2% (95% CI 83.5, 98.1), respectively, while the specificity was 97.1% (95% CI 84.7, 99.9) and 92.7% (95% CI 84.8, 97.3), respectively.

**Table 2:** Sensitivity, specificity, positive and negative predictive values of RDTs performed by CHVs compared to MLTs and segregated by gender

CHV vs MLT		
Characteristic	PE	(95% CI)
Overall		
Sensitivity	95.0	(87.7, 98.6)
Specificity	94.0	(88.0, 97.5)
Positive predictive value	91.6	(83.4, 96.5)
Negative predictive value	96.5	(91.2, 99)
Male		
Sensitivity	100.0	(83.9, 100)
Specificity	97.1	(84.7, 99.9)
Positive predictive value	95.5	(77.2, 99.9)
Negative predictive value	100.0	(89.4, 100)
Female		
Sensitivity	93.2	(83.5, 98.1)
Specificity	92.7	(84.8, 97.3)
Positive predictive value	90.2	(79.8, 96.3)
Negative predictive value	95.0	(87.7, 98.6)

*PE* point estimate; *CHV* interpretation by community health volunteer; *MLT* interpretation by medical laboratory technician

Overall sensitivity and specificity between CHVs performing RDTs and microscopy were 91.1% (95% CI 82.6, 96.4) and 88.8% (95% CI 81.6, 93.9), respectively (Table 3). Comparable performance in sensitivity and specificity were 91.6% (95% CI 83.4, 96.5) and 91.2% (95% CI 84.5, 95.7), respectively between MLTs performing RDTs and microscopy.

**Table 3:** Sensitivity, specificity, positive predictive value and negative predictive value, segregated by gender

Characteristic	Malaria cases (Pos vs Neg)			
	CHV vs microscopy		MLT vs microscopy	
	PE %	(95% CI)	PE %	(95% CI)
Overall				
Sensitivity	91.1	(82.6, 96.4)	91.6	(83.4, 96.5)
Specificity	88.8	(81.6, 93.9)	91.2	(84.5, 95.7)
Positive predictive value	84.7	(75.3, 91.6)	88.4	(79.7, 94.3)
Negative predictive value	93.6	(87.3, 97.4)	93.7	(87.4, 97.4)
Male				
Sensitivity	85	(62.1, 96.8)	86.4	(65.1, 97.1)
Specificity	91.2	(76.3, 98.1)	94.1	(80.3, 99.3)
Positive predictive value	85	(62.1, 96.8)	90.5	(69.6, 98.8)
Negative predictive value	91.2	(76.3, 98.1)	91.4	(76.9, 98.2)
Female				
Sensitivity	93.2	(83.5, 98.1)	93.4	(84.1, 98.2)
Specificity	87.8	(78.7, 94)	90	(81.2, 95.6)
Positive predictive value	84.6	(73.5, 92.4)	87.7	(77.2, 94.5)
Negative predictive value	94.7	(87.1, 98.5)	94.7	(87.1, 98.5)

*PE* point estimate; *CHV* interpretation by community health volunteer; *Microscopy* findings from the laboratory; *MLT* interpretation by medical laboratory technician

Comparing performance of diagnosing malaria by male and female CHVs with the MLTs, the sensitivity and specificity were 91.1% (95% CI 85, 96.8) and 91.2% (95% CI 76.3, 98.1) for males, respectively, and the sensitivity and specificity were 93.2% (95% CI 83.5, 98.1) and 87.8% (95% CI 78.7, 94) for females, respectively.

## Discussion

Many malaria endemic areas of the world lack sufficient capacity and resources for accurate diagnosis, and reliance on clinical symptoms and signs alone are inadequate and imprecise indicators of malaria disease. Use of CHVs has been shown to improve the acceptance of community interventions to address malaria diagnosis and treatment as CHVs are well respected within their communities of residence [20]. The age and gender characteristics of the participating CHVs were similar to those in a study by [21] which evaluated RDT use by community health workers.

This study demonstrated consistently similar results between RDTs conducted by CHVs and MLTs, and both results compared well with microscopy. A study done by [22], to compare RDTs and microscopy to diagnose malaria found 64% and 59% positivity of RDTs and microscopy, respectively. The difference could be because RDTs detect malaria antigens still in circulation after recovery from the disease, giving false positive results, as compared to microscopy that detects parasite forms.

Results of kappa scores, and sensitivity and specificity in this study were consistent with those of [21] which demonstrated that CHVs generally adhered to testing procedures, could safely and accurately perform RDTs, and interpreted test results correctly. While the findings contributed to the body of evidence that CHVs perform RDTs at an acceptable level [23], their skills were observed to improve with increasing years of experience. These findings could be as a result of routine support supervision and a good understanding of CCMm leading to its effective implementation.

Inter-rater reliability is important as it represents the extent to which the collected data in the study correctly represented the variables measured. The kappa statistic was used to test inter-rater reliability between the CHVs and MLTs. As guided by [19], the study kappa score was above 80% agreement, which is the minimum acceptable inter-rater agreement, and an indication for an almost perfect agreement between CHVs and MLTs.

CHVs achieved very good sensitivity, specificity, positive predictive values and negative predictive values. These results are similar to those reported in most studies although they used microscopy as the gold standard for diagnosis [23].

The difference in malaria positivity rates as reported in the KHIS needs to be studied further to understand the root cause of the difference. The national malaria surveillance data may need to be disaggregated by CHVs and health facilities at different levels, with follow-up of the different sources of data for verification.

## Conclusion

The ability of CHVs to diagnose malaria cases under the CCMm project compared well with the findings of qualified, experienced laboratory staff as evidenced by comparable sensitivity, specificity and kappa scores. CCMm should continue to scale up its valuable and important role of first-line diagnosis and treatment of uncomplicated malaria. Alternative possible causes of differing malaria positivity rates between those from CHVs and general national data need to be explored.

## Data Availability

The datasets used and/or analysed during the current study are available from the corresponding author on reasonable request.

## Abbreviations

AL: Artemether lumefantrine
CCMm: Community case management of malaria
CHC: Community health committees
CHEW: Community health extension worker
CHV: Community health volunteer
CI: Confidence interval
CU: Community units
IQR: Interquartile range
KHIS: Kenya Health Information System
LDH: Lactate dehydrogenase
MLT: Medical laboratory technician
RDT: Rapid diagnostic test
ODK: Open Data Kit
PfHRP2: *Plasmodium falciparum*-specific histidine-rich protein 2
RA: Research assistant
TPR: Test positivity rate
WHO: World Health Organization
